# Range quality assurance measurements for clinical and FLASH proton beam therapy using the quality assurance range calorimeter

**DOI:** 10.3389/fonc.2025.1622231

**Published:** 2025-11-21

**Authors:** Saad Shaikh, Sonia Escribano-Rodriguez, Raffaella Radogna, Ruben Saakyan, Sam Manger, Nicholas Henthorn, John-William Warmenhoven, Michael Taylor, Simon Jolly

**Affiliations:** 1 Department of Medical Physics and Biomedical Engineering, University College London, London, United Kingdom; 2 Department of Physics and Astronomy, University College London, London, United Kingdom; 3 Department of Physics, University of Bari, Bari, Italy; 4 Istituto Nazionale di Fisica Nucleare, Sezione di Bari, Bari, Italy; 5 Division of Cancer Sciences, Faculty of Biology, Medicine and Health, The University of Manchester, Manchester, United Kingdom; 6 The Christie NHS Foundation Trust, Manchester, United Kingdom

**Keywords:** proton therapy, FLASH, range quality assurance, photodiode, plastic scintillator

## Abstract

**Objective:**

This work demonstrated the design and performance of a full-sized clinical prototype of the Quality Assurance Range Calorimeter (QuARC): a segmented large-volume scintillator-based detector for fast, accurate proton range quality assurance (QA) measurements.

**Materials & methods:**

The detector used 128 scintillator sheets of size 105 × 105 × 3 mm arranged into 4 modules of 32 sheets, where each sheet was directly coupled to a photodiode. Fast analogue-to-digital conversion facilitated measurement of scintillator sheet light output to 20-bit precision at 6 kHz, with a dynamic range of up to 350 pC.

**Results:**

Proton range measurements with the full-size detector were performed at the proton therapy research facility based at The Christie at clinical dose rates, corresponding to ∼1 nA nozzle current, where the range accuracy of the QuARC was found to be within 0.4 mm of facility reference across the full clinical energy range. The QuARC successfully performed range measurements of the 245 MeV beam at FLASH dose rate (∼50 nA nozzle current), where the fitted range agreed with the clinical current measurement to 0.3 mm. Preliminary results show charge linearity of the detector to be within 3%.

**Conclusions:**

The QuARC has been shown to be a promising candidate for fast, accurate range QA at conventional clinical dose rates and thanks to its high precision and dynamic range, has been shown to also be viable at FLASH dose rates. Future work will investigate improving the accuracy and stability of the calibration process by optimising the scintillator sheet light output and mechanical setup.

## 1 Introduction

The superior dose conformity possible with Proton Beam Therapy (PBT) over conventional therapy necessitates more rigorous quality assurance (QA) procedures to ensure accurate dose delivery and optimal patient safety. Amongst the beam QA measurements carried out to ensure safe clinical delivery is a verification of the proton range in water, which is measured daily as part of standard clinical procedures (1). However, current methods for daily QA can be time-consuming and labour-intensive, with existing commercially available systems compromising between speed and accuracy. In particular, due to time constraints, daily range QA is normally limited to a small subset of the available beam energies used in the clinic, often only to verify beam intensity at a few prescribed depths. More comprehensive measurements of the full depth dose curve at multiple energies are reserved for monthly or yearly QA due to the time required for detector setup and measurement.

Over the last decade, FLASH proton therapy has received a large amount of interest as a potential paradigm shift in the way PBT treatments are delivered (2, 3). Pre-clinical evidence has been growing that full single-field dose delivery within a few hundred milliseconds, with dose rates above 40 Gy/s, can offer improved healthy tissue sparing by a factor between 1.1–1.8, whilst maintaining equivalent tumour control (4, 5). While there has been rapid growth in FLASH proton therapy research, there are still many unanswered clinical and technological questions — such as the exact dose rate needed for clinical treatments and the method of beam delivery — before FLASH treatment can be realised (6, 7). Amongst these are significant challenges with respect to dosimetry for FLASH proton therapy that must be overcome (8, 9). Of particular relevance to this work is that the gold-standard ionisation chamber-based detectors used for the measurement of proton depth-dose curves do not scale up to FLASH dose rates, largely due to the ion-recombination effect (10).

In the absence of an ideal system for daily range QA, research is underway to realise a plastic scintillator-based detector for fast, accurate measurement of proton range. Scintillators are of interest for proton range measurement due to their low cost, water equivalence, fast response times and dose rate independence (11). The latter two characteristics are of particular interest for FLASH proton therapy, since this provides the possibly of detectors that can operate at both conventional and ultra-high dose rates. A novel range telescope based around segmented polystyrene plastic scintillator sheets has been developed to improve the efficiency of range measurements in clinical PBT. A prototype system was constructed for QA measurements of pristine and spread-out Bragg peaks (SOBPs) at the Clatterbridge Cancer Centre (CCC) optical treatment room (12). This detector, tailored in size to the 60 MeV proton beam at the CCC, was able to measure the range of pristine Bragg peaks and SOBPs accurate to 0.2 mm, in a compact, self-contained package that could be mounted directly onto the proton nozzle. Building on this detector principle, this work explores expanding the device design to accommodate the full clinical energy range up to 250 MeV, whilst maintaining modularity and measurement speed, and investigating its applicability for FLASH proton therapy range QA measurements.

## 2 Background

While there is no universally enforced standard for proton QA procedures, there are generally agreed upon guidelines and prescriptions for which measurements should be acquired and the frequency of these measurements (1). Most modern PBT centres utilise pencil-beam scanning delivery systems, where daily checks are required for proton range and spot position, with range uniformity (i.e. range at different spot positions), full depth-dose curve and spot shape measurements performed on a yearly basis. However, in practice facility-specific experiences tend to inform specific protocols and many centres rely on procedures developed in-house for performing routine QA measurements (13). Given that FLASH PBT has not been fully realised in the clinic, there are currently no prescribed QA practices that are specific to FLASH in addition to those normally utilised for conventional dose rates.

### 2.1 Proton range quality assurance

Commercially available devices for proton range QA are typically ionisation chamber-based detectors, notably the PTW Peakfinder (14), and IBA Giraffe (15) and Zebra (16) detectors. The Peakfinder uses a small parallel-plate ionisation chamber submerged in a water tank and attached to a stepping motor to sample the proton depth-dose curve in down to 0.1 mm steps. As such, it demonstrates excellent spatial resolution, at the cost of slow measurement times — up to several minutes for a single depth-dose curve — meaning that full daily range QA would take up to an hour. For this reason, Peakfinders are rarely used for this purpose but are instead reserved for high precision pristine Bragg curve measurements at less frequent intervals. The IBA Giraffe/Zebra detectors on the other hand are Multi-Layer Ionisation Chambers (MLICs) that use a stack of ionisation chambers sandwiched between aluminium beam degrader plates. These devices can perform instantaneous measurements of proton depth-dose curves and, after fitting the measured curve with the Bortfeld model of the Bragg curve (17), have spatial accuracy of around 0.5 mm (18). While the Giraffe/Zebra demonstrate good accuracy, measurement stability and dose rate independence (in clinical current regimes) (19), the use of aluminium beam degrader plates loses water-equivalency. More importantly, device setup is not trivial and takes up valuable QA time: these systems can suffer from a lack of robustness requiring both detector and cabling to be handled with a degree of care that is suboptimal for a busy clinical environment.

For daily range QA, it is more common to use a device such as the IBA Sphinx and Lynx in conjunction: the former is a custom set of absorbers whilst the latter employs a large-area scintillator screen to image the 2D beam profile perpendicular to the beam axis (20, 21). This does not measure the full depth-dose curve but instead infers proton range by measuring proton beam spot intensity after passing through a set of absorbers of known thickness. This requires comparison against previous, more accurate measurements made with other devices (22–24). Therefore, while this device is easy to use and produces fast results, range accuracy is worse than other methods that directly measure the proton depth-dose distribution. Another device used for full daily QA is the Sun Nuclear Daily QA 3 (25), which consists of an array of diodes and ionisation chambers for measurement of a variety of beam parameters. Similar to the Lynx/Sphinx, this device performs indirect measurements of proton range for comparison against a previously measured baseline (26). As such, commercially available systems all exhibit drawbacks that compromise the ability to make accurate, online measurements of proton range for all proton energies within the time available for clinical daily QA. The relevant characteristics of current commercial offerings for daily range QA are summarised in **Table 1**.

**Table 1:** Summary of properties of commercial daily QA devices.

Device name	Detection technology	Range accuracy (mm)	Full depth-dose measurement?
PTW Peakfinder	Ionisation Chamber	±0.1 (27)	Yes
IBA Giraffe	Ionisation Chamber	±0.4 (18)	Yes
IBA Zebra	Ionisation Chamber	±0.4 (18)	Yes
IBA Lynx/Sphinx	Scintillation Screen & Camera	±0.5 (23)	No
Sun Nuclear DQA3	Ionisation Chamber & Diodes	±0.5 (26)	No

A more rigorous evaluation of daily QA practices can be found in (13).

The motivations for developing a scintillator-based detector for proton range QA and recent technological developments in this space have already been discussed in (12). In short, scintillator-based systems potentially offer attractive benefits such as reduced weight and costs and improved simplicity and robustness. Despite challenges with ionisation quenching, where the scintillation light output becomes non-linear with energy deposition in regions of high Linear Energy Transfer (LET) (eg. the Bragg peak), scintillator systems have been shown to reliably capable of measuring proton range (28, 29). There have been several bespoke detector systems built using volumetric scintillator for proton QA and imaging measurements over the last two decades (30, 31), however none have been made widely available commercially with specific focus on rapid QA for all clinical proton energies.

### 2.2 FLASH QA adaptations

When considering QA for FLASH PBT, ionisation chambers have the main drawback of showing dependence on dose rate due to volume ion recombination, which causes the charge collected in the chamber to become non-linear with high spatial densities of ionised particles (10, 32). As a result, the commercial devices listed above cannot be used for dosimetry at FLASH dose rates. One notable exception to this is the PTW Advanced Markus chamber which has been successfully used with high dose rate electron beams, using an empirical model to correct for the ion-recombination effect (33). Some recent works have also shown that plane-parallel ionisation chambers with small electrode spacing demonstrate good linearity with dose rate (34–36), as well as correction methods other than empirical models (37), but significant work is still required to realise general-purpose commercial devices for proton FLASH dosimetry.

While the primary focus of this work is to expand the design of the QuARC to accommodate the full clinical energy regime, organic plastic scintillators are also of interest for FLASH QA (38). The most significant benefits are the fast response times on the order of nanoseconds (39) and dose rate independence in the scintillation light output (40, 41). The Exradin W2 scintillator has already demonstrated some success with ultra-high dose rate dosimetry with electron beams (42). However, it should be mentioned that some works have demonstrated dose rate dependence with scintillators that require polynomial corrections (43), suggesting that the method of light collection is critical.

## 3 Materials and methods

The Quality Assurance Range Calorimeter (QuARC) is a series of optically-isolated polystyrene scintillator sheets that measure proton range by sampling the scintillation light emission of a proton beam in the scintillator volume. The segmented design allows instantaneous, direct measurement of the light output of each scintillator sheet individually, with each sheet coupled to a photodiode: the measured light levels are then used to create a coarse depth-light distribution. This is then fitted to an analytical depth-light model, where the proton range is a free fit parameter and the proton depth-dose curve can be reconstructed. The QuARC therefore seeks to achieve the speed needed for daily QA while enabling the reconstruction of full depth-dose distributions, providing superior accuracy than methods that merely infer proton range.

### 3.1 Detector design motivation

Alongside the original motivation for developing a scintillator-based proton range QA system set out in (12), the primary design goals for the updated QuARC were as follows:

(i) Real-time online range measurement. Whilst the exceptionally fast response time of plastic scintillators is critical, this also necessitates a fast light readout and Data Acquisition (DAQ) system that can provide real-time display of the proton range as the beam is being delivered.

(ii) Coverage of the full range of clinical energies, from 70–250 MeV, without reduction in the resolution of the range measurement.

(iii) Minimal weight and footprint to improve detector handling. A scintillator volume in the detector of some 4 litres corresponds to a mass of ∼4 kg, potentially enabling a complete range detector of less than 5 kg. This is considerably lighter than equivalent MLIC-based systems could achieve. Light weight retains the option for a nozzle-mounted system, such as that realised for the Clatterbridge detector, simplifying setup.

(iv) Ease of use and setup. In packaging the digital detector electronics within the detector housing, cabling can potentially be reduced to a single power cable with an optional network cable where wireless networking is not available.

(v) Improved detector robustness. In keeping to a modular detector design within a robust enclosure, the detector can be better insulated from the rigours of daily clinical use. In addition, detector maintenance is simplified since scintillator modules can be quickly exchanged should the electronics fail or scintillator burn out.

(vi) Simple system control without the need for bespoke software installation and without restricting the detector or display capability. On-board DAQ enables a full web-based interface allowing any device with a web browser to be used for detector control.

### 3.2 Scintillator range telescope

A number of improvements were made to the system described in (12) in order to meet the challenges of measuring the full range of clinical proton energies. The full-sized version of the QuARC uses 128 scintillator sheets, each 105 × 105 × 3 mm, arranged into 4 modules of 32 sheets, for a total detector depth of approximately 384 mm. The scintillator (manufactured by NUVIATech Instruments) has peak emission at 425 nm, refractive index of 1.57, density of 1.03 g/cm^3^, light output 56% of anthracene and decay constant of 2.5 ns (44). The detector dimensions were selected in order to fully contain a clinical proton pencil beam at 250 MeV, the maximum energy delivered by the commercial PBT systems currently available [see **Table 1** in (7)]. The transverse dimensions correspond to a Water Equivalent Thickness (WET) of ∼108 mm, large enough to fully contain the transverse spread of a clinical proton pencil beam up to 250 MeV. The combined longitudinal WET of the 4 modules is ∼395 mm, exceeding the proton range in water at 250 MeV of ∼380 mm and containing the complete Bragg curve distal fall-off.

**Figure 1:**
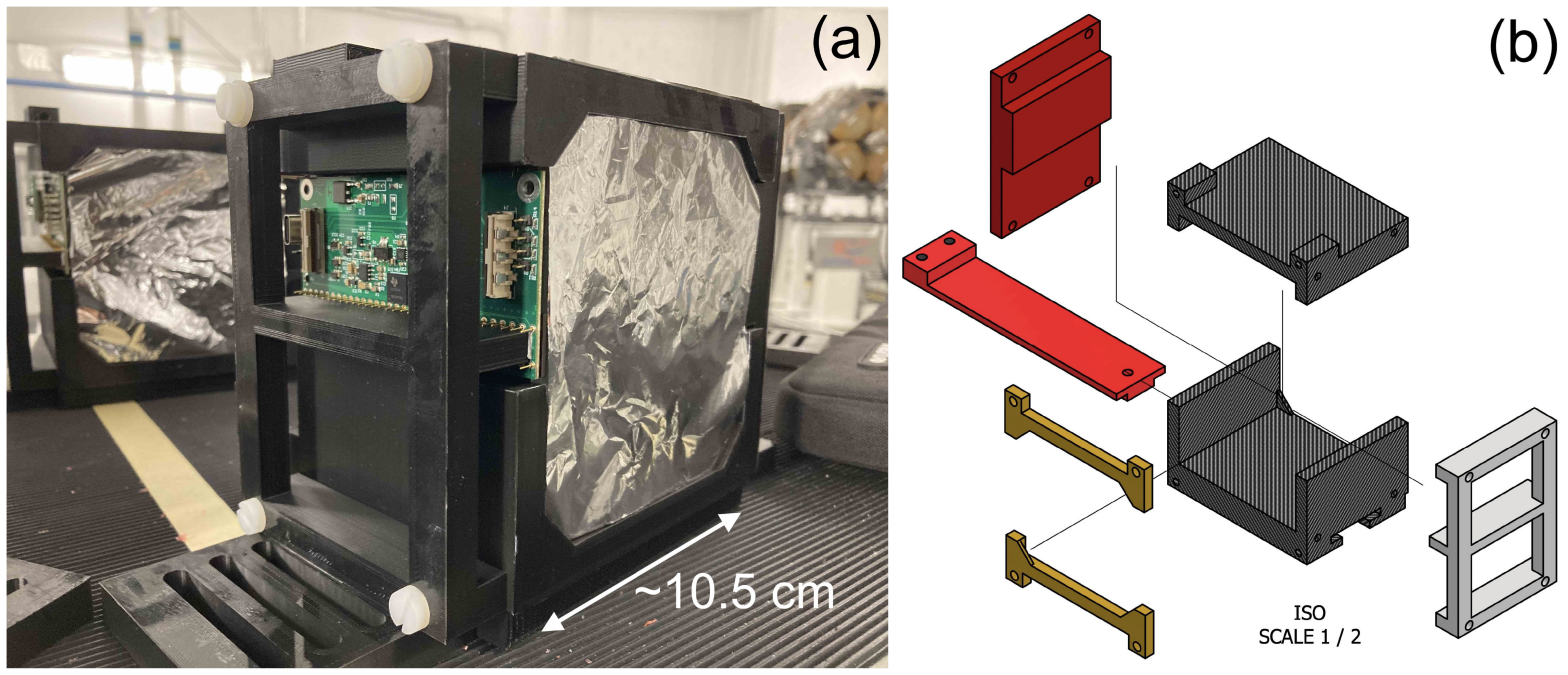
QuARC detector module design showing **(a)** single 32-sheet module and **(b)** stack holder CAD schematic.

Each of the scintillator stacks that make up the 4 detector modules is housed in a custom 3D-printed frame made up of black polylactic acid (PLA) that securely holds the 32 scintillator sheets together. An assembled detector module can be seen in **Figure 1a** and specific details about each detector module can be found in section 4.1. Thin sheets of 6 µm thick aluminised Mylar foil, matched to the transverse dimensions of the scintillator, are sandwiched between each scintillator sheet to provide optical isolation. An extra sheet of foil is placed at the front and back of each detector module to prevent cross-module light leakage. This stack of scintillator and aluminised Mylar is then held in place within the 3D-printed frame by a pair of 3D-printed clamping bars at the top and bottom of the sheets, as shown in **Figure 1b**. The steps on the frame and the clamping bars are 3 mm thick, resulting in a 6 mm air gap between scintillator modules when they are placed with the largest faces in contact. Given the 3 orders of magnitude smaller WET of air compared to the scintillator, this small gap makes an extremely small contribution to the depth-dose curve that is straightforward to account for in the reconstruction. The four detector modules are then assembled end-to-end, forming the complete range telescope with a physical thickness of 402 mm: 384 mm coming directly from the scintillator sheets and the remainder from the 6 mm air gaps between modules. Further details of the full stack assembly are shown in Section 3.4. Each module has a slot in the base for a support rail to which the module is mounted: this enabled rapid installation of the modules as well as a variety of mounting options depending on experimental setup requirements.

**Figure 2:**
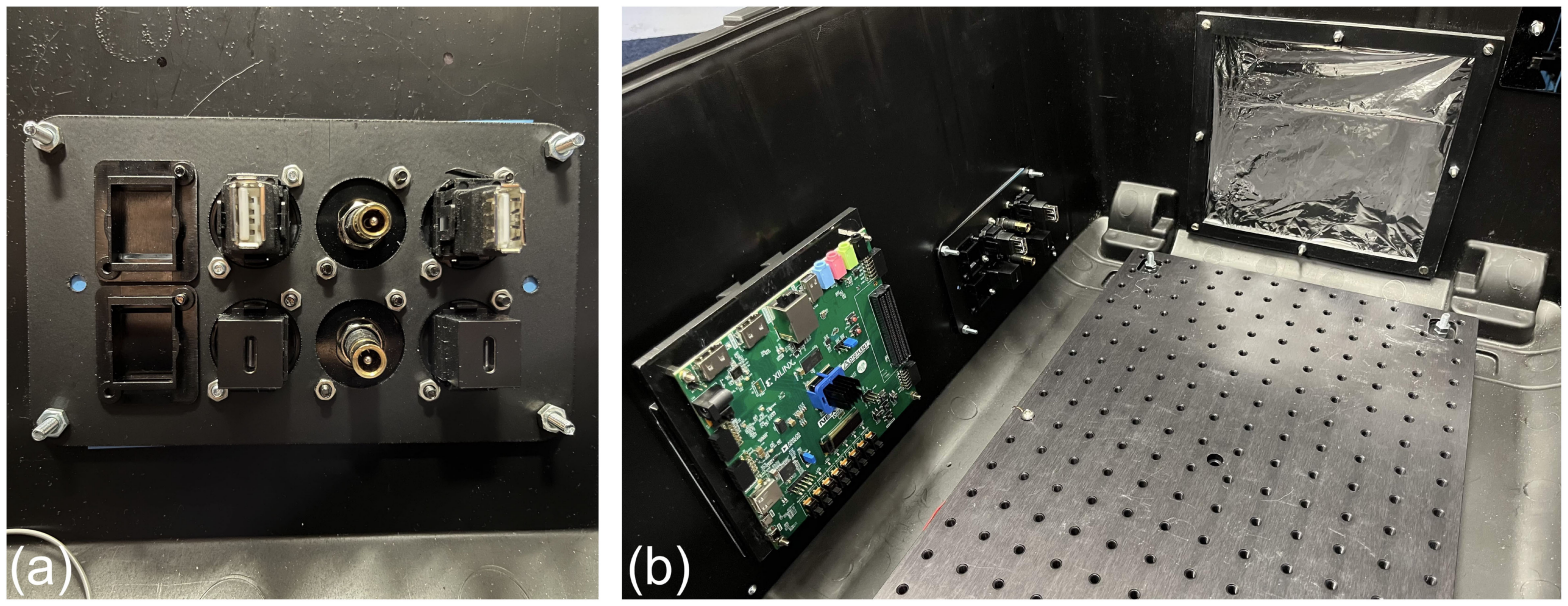
QuARC experimental detector enclosure showing **(a)** customisable XLR patch panel for FPGA data and power connections and **(b)** internal view with the mounted FPGA (left), data/power patch panel (middle), Mylar window (right) and optical breadboard base plate.

The scintillator modules are housed in an experimental detector enclosure constructed from a PELI 1637 AIR light-tight flight case (45): this was then modified to enable easy transport and rapid deployment of the detector. A ThorLabs MB3045/M optical breadboard (46) was bolted to the internal base of the case: with threaded M6 bolt holes on a 25 mm pitch, multiple detector mounting positions and arrangements could be tested by bolting the mounting rails in the appropriate position. With the optical breadboard aligned to the inside of the case, easy alignment of the scintillator modules relative to the detector enclosure was possible. Throughout the scintillator module assembly and detector enclosure, nylon bolts were used to minimise beam scatter, with the exception of the bolt securing the breadboard to the inside of the case. Holes were cut in the long sides of the case and patch panel plates installed to allow detector signal and power connections to pass through to the internal electronics. These patch panels were based around 2-gang 8-port XLR patch panel blanks, allowing custom connectors utilising the XLR standard to be mounted and removed as needed: this is shown in **Figure 2a**. A custom mount for the FPGA used for detector control and acquisition was 3D-printed, allowing the FPGA to be secured to the inside wall of the case. In addition, square holes ∼ 200 × 200 mm were cut into each end face of the case for beam windows: a Mylar foil window (also 6 µm thick) was installed at each end of the case to facilitate light-tight beam transport into the detector enclosure, with little beam spread. The resulting interior of the enclosure is shown in **Figure 2b**.

### 3.3 Data acquisition and processing

**Figure 3:**
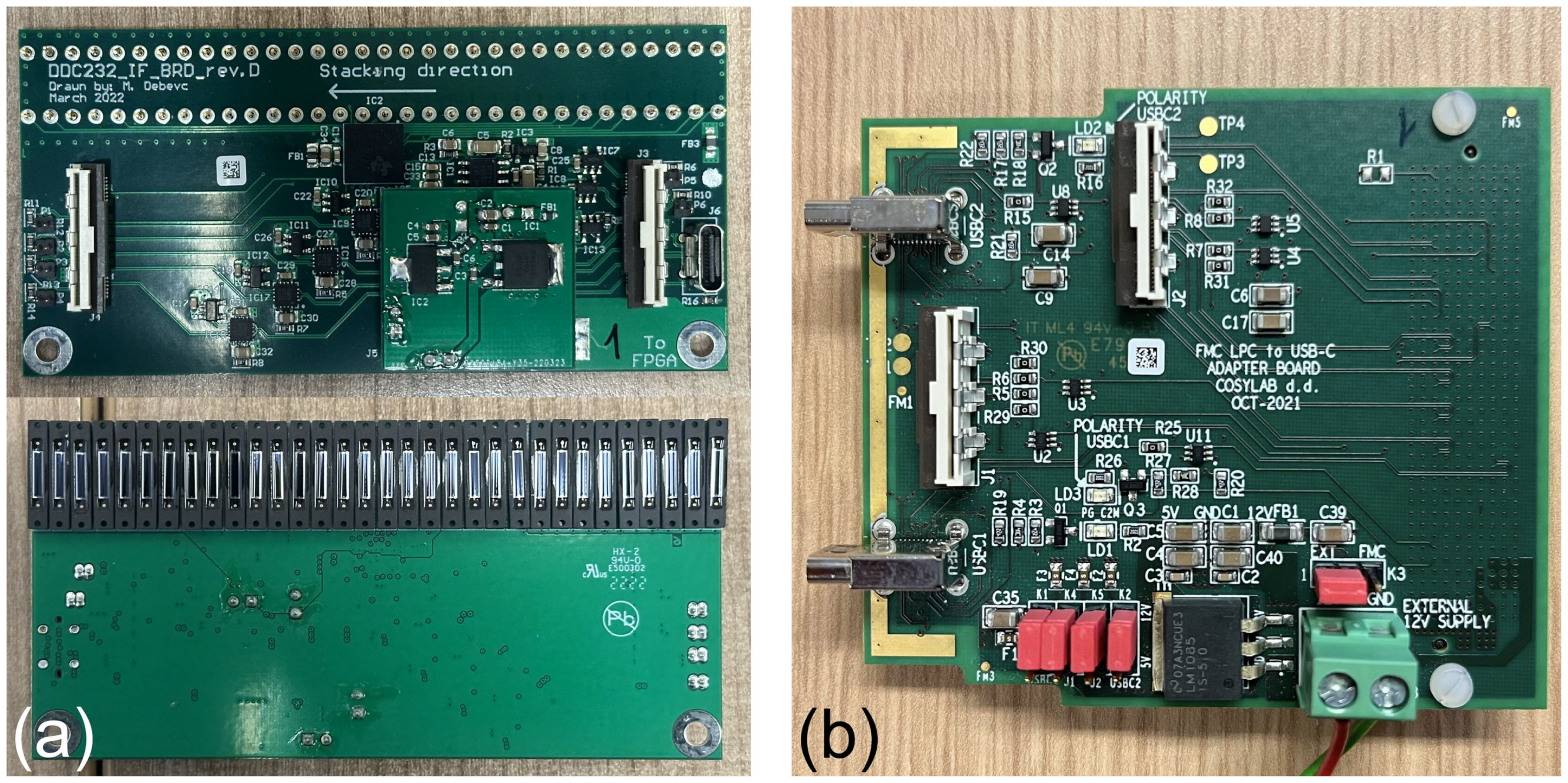
QuARC front-end electronics showing **(a)** DDC232 circuit board and **(b)** FMC daughter board.

Each detector module has a custom designed circuit board manufactured by CosyLab that houses 32 Hamamatsu S12915-16R photodiodes spaced 3 mm apart and a Texas Instruments DDC232-CK analogue-to-digital converter (ADC) (47). The DDC232 (**Figure 3a**) is a low-power 32-channel ADC used to perform photodiode charge integration and features zero-deadtime dual-integrators with integration times between 166.5 µs–1s, 20-bit precision and 8 assignable full-scale ranges (FSR) from 12.5–350 pC. Each photodiode is connected to a single DDC232 input and DDC232 chips can be daisy-chained to expand the number of readout channels: this enabled all of the front-end electronics to be read out together in a single readout cycle. The DDC232 circuit boards are interfaced to a Digilent Nexys Video development board (48) via a custom CosyLab daughterboard (**Figure 3b**) that uses the low-pin count FPGA mezzanine card (LPC FMC) to provide both 7.5 V power and data signals to the DDC232 boards: the 7.5 V power is supplied through an external power supply connected to the FMC through a 2.5 mm DC barrel jack. Connection between the daughterboard and the DDC232 and between daisy-chained DDC232 boards are facilitated using Molex ribbon cables, where only the last DDC232 board in the chain needs to be connected to the daughterboard A custom mount for the FPGA was also 3D printed to enable it to be securely mounted and easily removed from the inside of the detector enclosure.

DDC232 configuration and data acquisition is performed using a laptop running a custom C++ script that interfaces with the FPGA via USB 2.0 (49). In short, operation involves the PC sending commands to set the user-assigned DDC232 integration time and FSR parameters, as well as the number of DDC232 boards in the chain and the total number of measurements to acquire. Digitised photodiode integration results are shifted along a serial data line each integration cycle and then transferred to the PC. In addition to saving raw data, the software back-end provides a live display of the photodiode charge levels at 50 Hz and can perform real-time depth-dose curve fitting for live range reconstruction at up to 5 Hz. Detector calibration requires a background measurement and “shoot-through” measurements which accounts for differences in individual scintillator sheet light output, photodiode response and scintillator-photodiode coupling. This process is performed for each detector module in the chain individually and is described in detail in (12). The Kelleter model (29), which is an analytical model that describes proton depth-light distributions by applying the empirical Birks’ law for scintillator light quenching (50) to the analytical Bortfeld description of the Bragg curve (17), is used to perform function fitting on the calibrated scintillator sheet light output. The proton range is a free fit parameter and the Bortfeld depth-dose curve can be easily recovered from the fitted result by setting the quenching parameter, Birks’ constant (*kB*), to zero.

### 3.4 Detector characterisation

An experiment was conducted at the proton therapy research facility at The Christie Hospital (Manchester, UK) to benchmark the range accuracy of the QuARC with proton beams across the full clinical energy spectrum. The Varian cyclotron at The Christie is commissioned to produce a 250 MeV proton beam where, after energy selection, a 245 MeV beam with an instantaneous FLASH dose rate of up to 112.7 Gy/s and nozzle current of up to 56.4 nA can be delivered (51). This enabled a preliminary investigation of the performance of the QuARC with FLASH proton beams. As with all clinical cyclotron-based PBT systems, an energy degrader is used to reduce the beam energy from the fixed 245 MeV cyclotron extraction energy, resulting in a significant variation in transported current across the clinical energy range. This meant that the currents needed to achieve FLASH dose rates were only achievable for the highest energies. In addition, the Varian control system provides control over the extracted ion source current, rather than the actual nozzle current, since for a clinical system it is the total dose that is most critical, not the instantaneous current. As such, the current values quoted are for the extracted ion source current.

**Figure 4:**
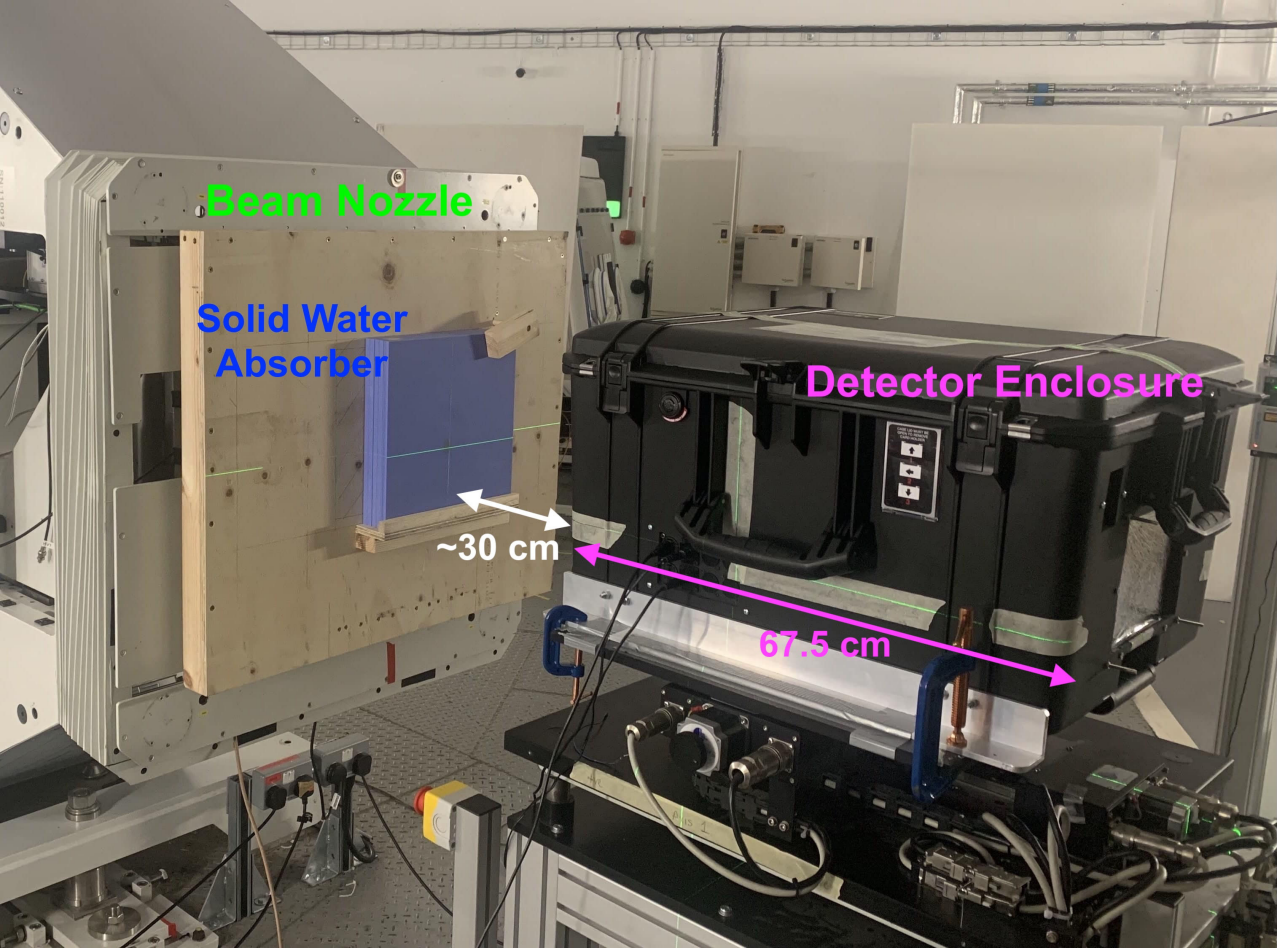
Experimental enclosure placed on an adjustable table in front of the horizontal research beamline at The Christie.

The research room at The Christie uses a horizontal fixed beamline that terminates in a Varian engineering nozzle (51). The detector enclosure was mounted onto an experimental table with 3D position and tilt adjustment with the upstream entrance window of the enclosure 30 cm from the exit window of the nozzle. The scintillator modules were placed a further ∼10 cm deep in the enclosure with the upstream face of the detector module aligned to the research room focal plane using the in-room laser alignment system: this is shown in **Figure 4**.

**Figure 5:**
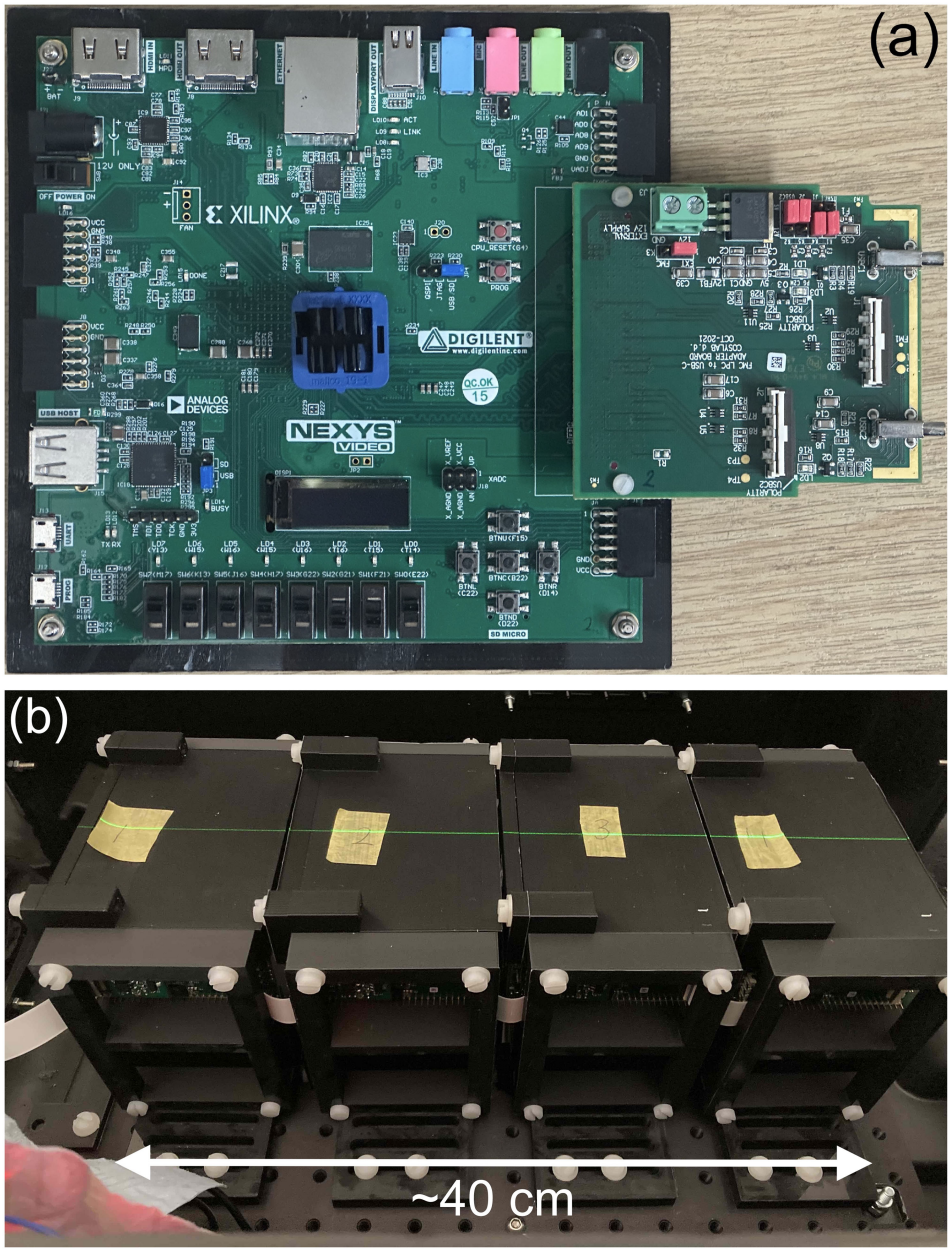
Detector setup showing **(a)** the FPGA connected to the FMC daughter board and **(b)** four modules daisy-chained in the experimental enclosure.

**Figure 5a** shows the custom FMC daughter board from **Figure 3b** connected to the Nexys Video FPGA development board. A single Molex ribbon cable is then connected to the first of the four detector modules connected in series, shown in **Figure 5b**. The DAQ FPGA was controlled over USB by an in-room MacBook Pro running the necessary C++ DAQ code; this in turn was controlled over a network through a remote desktop connection, allowing full DAQ control from the research control room. For all measurements, a DDC232 integration time of 170 µs was used, with the FSR adjusted according to the beam current. Each run recorded 30,000 photodiode measurements, corresponding to around 5 s of exposure.

#### 3.4.1 WET measurements

While plastic scintillator has a very similar density to water, the physical scintillator depth must be accurately calibrated to Water-Equivalent Thickness (WET) in order to achieve sub-mm range accuracy. To do this, a range pullback test was performed using the PTW 34070 Bragg Peak chamber in a PTW MP3-M water phantom (52). The range pullback test measured the WET of each detector module by using the ionisation chamber to measure the depth-dose curve of the highest energy proton beam available both with and without the detector module under study placed upstream. The difference in the measured range provides the WET of the module and its relative stopping power (RSP) can then be found by dividing the modules’ measured WET by its physical thickness. The RSP is used to convert the thickness of each scintillator sheet to WET in the measured proton depth-light curve. For the QuARC scintillator module WET calibration, a 245 MeV proton beam at clinical current (∼18 nA ion-source current) was used for range pullback tests of each of the four detector modules, as well as the empty detector enclosure. The latter measurement was performed to determine if there was any measurable systematic range offset from the enclosure itself.

#### 3.4.2 Detector calibration

To calibrate the detector for a range measurement, background and shoot-through measurements are required. The shoot-through measurements are used to calibrate for differences in individual scintillator sheet light output, photodiode response and scintillator-photodiode coupling: this is discussed in (12) and (28). For the shoot-through calibration, a 245 MeV proton beam (18.8 nA ion source current) was shot through the front and back of each 32-sheet detector module in turn, with the module rotated by 180 degrees between exposures to allow the beam to pass through the stack in the opposite direction. The resulting shoot-through light output from both detector orientations was then averaged, which provides a flat depth-light distribution with minimal scintillator light quenching, and thus a correction factor that can be applied to a measured depth-light curve to remove inconsistencies in the light output and coupling of each scintillator sheet. A ∼50 mm solid water absorber was placed upstream of the detector during shoot-through measurements to remove the small dose build-up at the start of the Bragg curve (53).

#### 3.4.3 Clinical range accuracy

Range measurements of pristine Bragg curves between 60–245 MeV in 10 MeV steps were performed at clinical currents to determine the range accuracy of the QuARC across the full clinical energy spectrum. The reconstructed Bortfeld depth-dose curves were then compared against facility reference data (measured using the PTW 34070 Bragg Peak ionisation chamber) to quantify the accuracy of the fitted range and the overall curve shape. The range measurements also serve as a benchmark for the RSP measurements from the range pullback tests, as an incorrectly calibrated RSP would result in an energy-dependence in the difference between the measured and facility reference range.

#### 3.4.4 FLASH performance

Given the large amounts of headroom available on the photodiode ADC, it was possible to investigate the detector response at nozzle currents up to 50 times that used clinically and determine if the reconstructed range was dependent on dose rate. Measurements of the depth-light curve and proton range were performed using the maximum beam nozzle current of 800 nA for 70, 150, 200 and 245 MeV proton beams. Additionally, as a preliminary investigation of the current linearity of the QuARC, measurements at 150, 200 and 245 MeV were taken at clinical current (386, 124 and 18.8 nA ion-source current respectively) and compared against the maximum ion-source current available of around 800 nA.

## 4 Results

### 4.1 WET measurements

The results of the range pullback tests are summarised in **Table 2**. The detector modules used sheets with sanded faces and polished edges, comparing two methods of sheet manufacture: injection moulded and machined block. The former uses a mould to inject liquefied scintillator before solidifying into a sheet; the latter cuts sheets from a larger block that is itself formed from liquefied scintillator that is poured into a large mould and pressed to the correct thickness. The main difference between these two methods is the production cost and sheet thickness accuracy, with the machined block method being more accurate but more expensive. From a batch of 73 machined block and 56 injection moulded sheets, the former had an average thickness and standard deviation of 2.96±0.036 mm whereas the latter was 2.92±0.072 mm, suggesting that the machined block method does indeed produce more consistent sheet thicknesses. In addition, non-uniformities were observed on the surfaces of corners of the injection moulded sheets from the injection moulding ports.

**Table 2:** WET Measurements of scintillator stacks.

Configuration	Sheet type	Thickness (mm)	WET (mm)	RSP
Stack 1 Front	Injection Moulded	95.95±0.05	98.24±0.10	1.024±0.001
Stack 1 Back	Injection Moulded	95.95±0.05	98.19±0.10	1.023±0.001
Stack 2	Machined Block	96.81±0.08	98.42±0.10	1.016±0.001
Stack 3	Injection Moulded	95.48±0.06	97.15±0.50	1.017±0.005
Stack 4	Moulded+Machined	95.07±0.05	94.67±0.50	1.006±0.010
Empty Box	N/A	0.012	0	N/A

The “empty box” configuration corresponds to the thickness of two sheets of Mylar foil (beam transport windows). The physical thickness uncertainty represents an average of three direct measurements of the stack at different positions. The uncertainty on the WET measurement reflects the sampling precision used in the PTW detector during the pullback experiment. These uncertainties are propagated in quadrature for the RSP value.

The uncertainty of the WET/RSP measurement increases for stacks 3 and 4 due to repeated beam drop-outs from the cyclotron that prevented proton beams from being delivered for sufficient duration to allow the PTW system to complete a measurement. The measurements for stacks 3–4 had to be completed with 0.5 mm spatial resolution around the Bragg peak rather than the standard 0.2 mm in order to reduce the number of sampling points and speed up the measurement, which reduced the precision of the measured Bragg curve. In particular for stack 4, only the Bragg peak itself was measured, corresponding to around 25% of the full curve. These RSP measurements were verified by investigating the relationship between beam energy and the resulting range accuracy, which is discussed further in section 4.3.

### 4.2 Detector calibration

**Figure 6:**
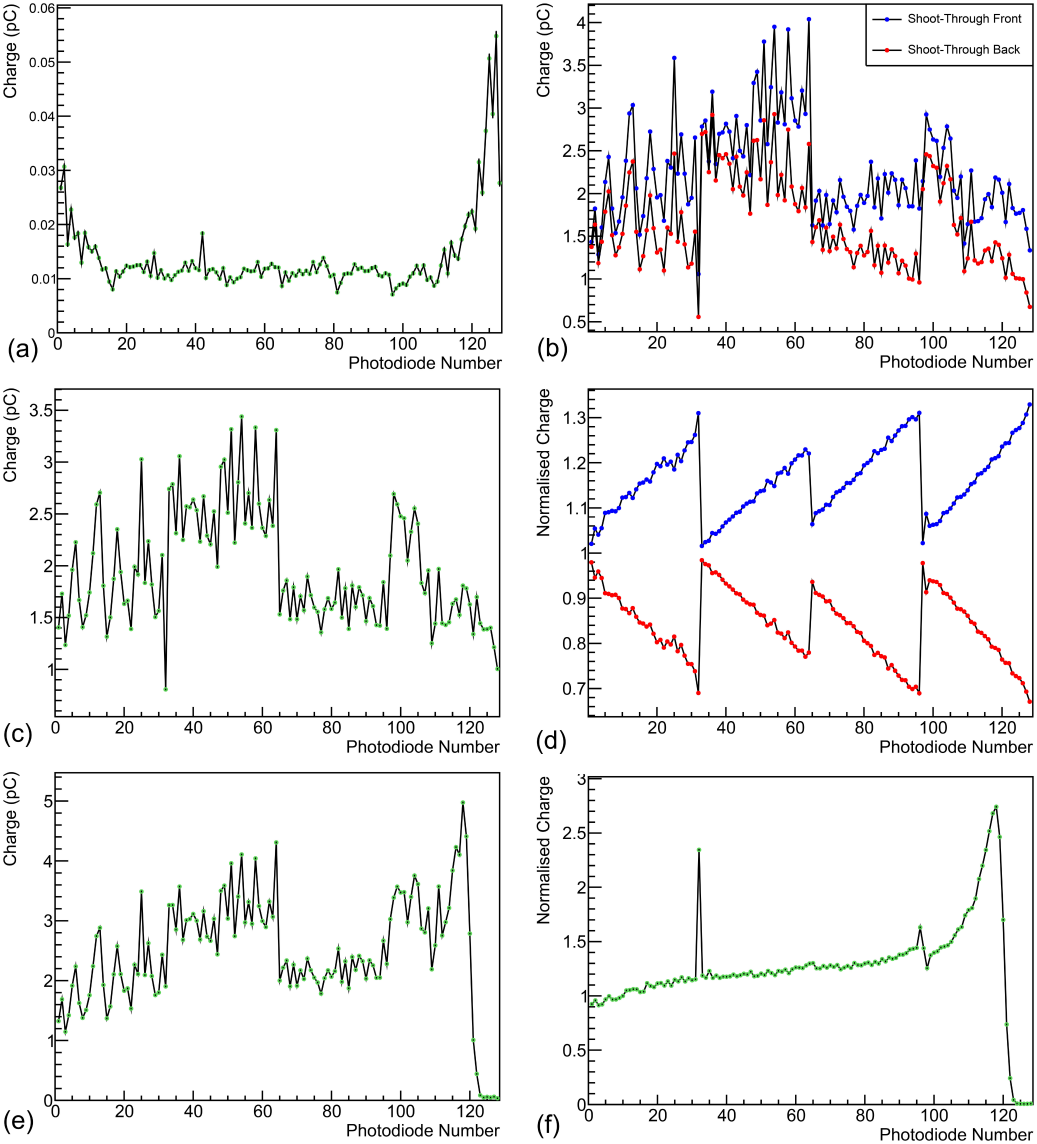
QuARC calibration measurements: **(a)** background, **(b)** front and back shootthrough calibration curves, **(c)** averaged calibration curve, **(d)** calibrated front and back shoot-through curves and **(e)** raw and **(f)** calibrated 245 MeV depth-light curves.

A background measurement was taken with the detector enclosure lid closed and room lights switched off: an average photodiode charge of 0.02 pC was measured, which can be seen in **Figure 6a**. The measured background increases for photodiodes at each end of the scintillator stack due to the closer proximity to the LED light leakage around the sheets caused by DAQ electronics within the enclosure (e.g. FPGA). The front and back shoot-throughs and the resulting calibration factors for each photodiode in the 4-module chain can be seen in **Figure 6b, c** respectively, with **Figure 6d** showing the application of the averaged calibration curve to each shoot-through, demonstrating the slope in each and therefore the need for averaging. The raw and calibrated depth-light curves for a 245 MeV beam at 18.8 nA ion-source current are shown in **Figure 6e, f** respectively.

It is apparent that even after calibration, the slope of the depth-light curve is not particularly smooth, there is significant stepping in the absolute light level from one module to the next, and there are 4 artefacts seen in photodiodes 32, 96, 97 and 98. The lack of smoothness in the slope and inter-module stepping is attributed to the sub-optimal surface finish quality of the scintillator sheets, which reduces the efficiency of photon propagation and internal reflection. It is likely that the individual photodiode artefacts were caused by changes in the photodiode-scintillator coupling between the calibration and range measurements, as the discontinuities in **Figure 6f** can be seen in **Figure 6d**. The range measurements were performed first after which the detector chain was disassembled for individual module calibration measurements. This required detector modules to be rotated/replaced within the detector enclosure and for the module circuit boards to be disconnected/reconnected. To compensate for this effect and allow range reconstruction, the calibration curve for photodiodes with artefacts (numbers 32, 96, 97 and 98) were manually adjusted to remove the observed discontinuities in **Figure 6d** by dividing with the following correction factors respectively: 2.05, 0.90, 0.94, 1.07. The same factors were used for all range measurements.

On average, the uncertainty on the calibrated photodiode charge levels is approximately 0.5%, after propagating the systematic DDC232 performance uncertainty (0.05%) and the statistical uncertainty from averaging 30,000 photodiode measurements. Given the fluctuation seen in the calibrated depth-light curve, it was apparent that the overall uncertainty was significantly underestimated and an estimation of the systematic uncertainty contribution from the (suboptimal) calibration process is required. The overall photodiode charge uncertainty after calibration was therefore raised by a factor of 20, preserving relative differences in photodiode uncertainties, such that the reduced $\chi^{2}$ in fitted curves (shown in section 4.4) across all energies were physical and informative of the overall goodness-of-fit.

### 4.3 Clinical range accuracy

**Figure 7:**
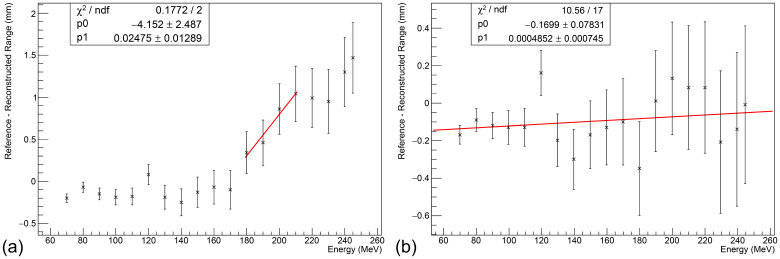
Relationship between beam energy and range accuracy at clinical currents. **(a)** Without adjustment, a clear linear trend is observed in stacks 3 and 4. **(b)** After increasing the RSP of stack 3 sheets to 1.029, no statistically significant trend is observed. An average systematic offset of about -0.15 mm is observed. Error bars calculated under the assumption of an optimal Δ*RSP* = 0.001, excluding flat contribution from the Kelleter model to isolate WET-related uncertainties.

**Figure 7a** shows the relationship between the range accuracy and beam energy using the RSPs found in **Table 2**. While the reconstructed range remains relatively consistent for modules 1 and 2, there is a sharp linear trend for modules 3 and 4, suggesting that the measured RSP of module 3 (and the sheets of those type in module 4) is ∼ 1.3% too low. Increasing the RSP of stack 3 to 1.029, with the associated sheets in stacks 4 being modified accordingly (see **Table 2**), provides the relationship shown in **Figure 7b**, where no statistically significant trend is observed. Across the energy range, the reconstructed range is accurate to better than 0.5 mm to the reference, with an average systematic offset of around -0.15 mm. The range uncertainty is calculated from three components added in quadrature:

(i) the systematic uncertainty of the Kelleter model (0.2 mm);

(ii) the systematic uncertainty on the WET from potential rotational misalignment of up to 2 degrees of the detector enclosure (*L*(1 − cos2°));

(iii) the systematic uncertainty on the RSP measurement itself (multiplied by the range).

With these three components, the range uncertainty was found to be 0.2 mm at 70 MeV and 0.5 mm at 245 MeV.

### 4.4 FLASH performance

**Figure 8:**
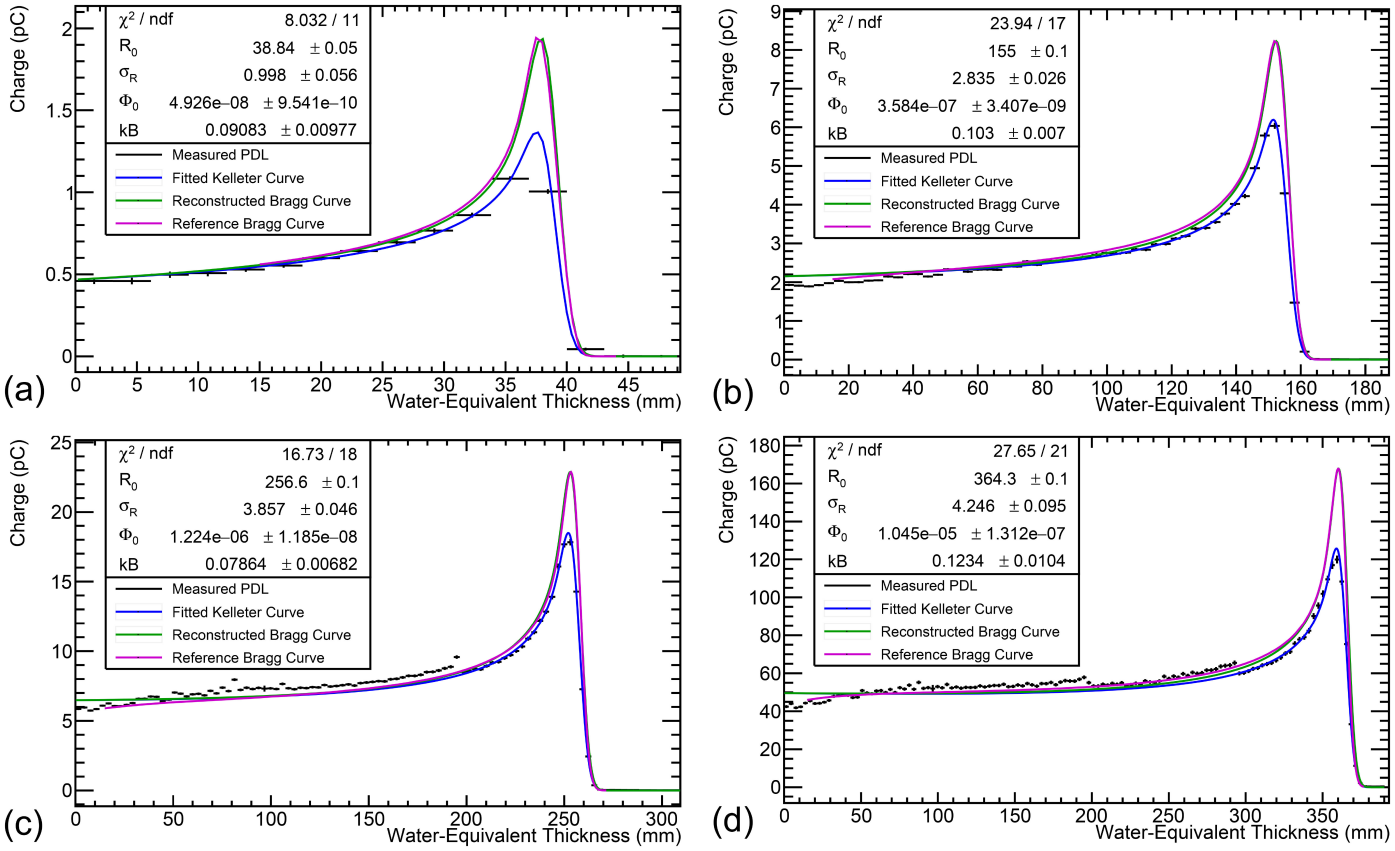
Fit results for **(a)** 70, **(b)** 150, **(c)** 200 and **(d)** 245 MeV beams at 800 nA, after RSP adjustment shown in **Figure 7b**. The width of the black horizontal bars in the Proton Depth Light (PDL) curve represents the WET of each scintillator sheet, with photodiode charge uncertainty as described at the end of section 4.2. The blue curve is the fitted quenched Bragg curve and the green curve is the reconstructed Bragg curve (i.e. kB=0). Only the fit uncertainty is shown in *R*_0_. The reconstructed Bragg curve is normalised to be equal to the quenched Bragg curve at 0 mm. The magenta curve shows the facility reference depth-dose curve and is normalised to match the height of the reconstructed Bragg curve. The fit range was limited to the module containing the Bragg peak, to mitigate the stepping observed in light levels across modules. An offset of 15mm was applied to the reference curve to account for systematic offsets in its measurement. The reference ranges are **(a)** 38.7 mm **(b)** 155.0 mm **(c)** 256.7 mm **(d)** 363.9 mm, all with uncertainty ±0.1 mm.

**Figure 8** shows the fit results for 70, 150, 200 and 245 MeV proton beams with a cyclotron ion source current of 800 nA after modifying the WET of stacks 2–4 as shown in **Figure 7b**, such that there is no significant dependence of the range reconstruction difference with energy. These illustrate the transmission current loss that occurs from stepping down the proton energy and the large dynamic range available with the DDC232, which is set accordingly for each measurement. Consequently, the 245 MeV beam has the largest current and can be considered a FLASH beam, whereas the 70 MeV beam has the smallest nozzle current and is the value used clinically.

**Table 3** shows the results of current linearity investigations using 150, 200 and 245 MeV proton beams at clinical and maximum currents. Since exact measurements of the nozzle current were not available during beam delivery, the ratio of the integral charge measured in the calibrated depth-light curve for the high and low currents was calculated and compared to the ratio of the beam ion-source current: since the proportion of transported current for a given beam energy/degrader setting is not dependent on the peak current, this provided a way of estimating the linearity of the detector. It should be noted that currents for conventional dose rate beams delivered to the research room at The Christie at and below 120 MeV use the full ion-source current of 800 nA and therefore cannot be used to investigate current linearity in this way.

**Table 3:** Current-charge linearity results using clinical and maximum beam currents.

Energy (MeV)	150	200	245
Clinical Ion-Source Current (nA)	386	124	18.8
FLASH Ion-Source Current (nA)	807	785	810
Beam Current Ratio	2.09	6.33	43.1
Integral Photodiode Charge Ratio	2.16±0.01	6.41±0.01	44.4±0.1
Ratios Percentage Difference	3.35%	1.26%	3.02%
Clinical-FLASH Fitted Range Diff. (mm)	0	0.1	0.3

The integral photodiode charge ratio is calculated by dividing the sum of all signal photodiodes for high and low currents. The uncertainty is calculated using the individual photodiode charge uncertainties.

The integrated charge ratio shows a small systematic discrepancy on the order of 1-3% between the beam current ratio, and at least within the limited data available, no apparent trend with energy is observed. No modifications were required to fitting parameters in the FLASH regime and the reconstructed value of Birks’ constant agreed within the fit uncertainty range. Additionally, any difference in the fitted range between the high and low current regimes remains within the uncertainty of the detector of a maximum of 0.5 mm at 245 MeV.

## 5 Discussion

### 5.1 Detector performance

The QuARC design was successfully expanded to incorporate 128 scintillator sheets by daisy-chaining multiple ADCs, without loss of speed or dynamic range, and thus perform range QA across the full clinical proton energy range. Thanks to the user-assignable dynamic range and 20-bit digitisation precision, the QuARC was comfortably able to perform measurements at clinical currents (approx. 1 nA) using the smallest FSR (12.5 pC) and scale up to FLASH currents of around 50 nA nozzle current using the largest FSR (350 pC). The main limitation with this prototype stems from the use of unpolished scintillator, which has been shown to worsen the shape of the calibrated depth-light curves, especially when compared to previous work using polished scintillator (12, 28). Issues with calibration across multiple modules could also be attributed to this, however could also be due to a fundamental limitation of the calibration process, which may prevent translating the shoot-through process across multiple modules. This is due to the changing spread of the proton beam with depth when calibrating multiple modules, though this requires further investigation. Nevertheless, despite these calibration issues, the QuARC was still able to consistently reconstruct the range of both clinical and FLASH beams correct to within 0.5 mm by restricting the fit range to the module containing the Bragg peak. This accuracy is well within the requirements of a clinical device and is competitive with other commercial offerings.

### 5.2 Clinical applications

The full-size version of the QuARC presents a viable, attractive alternative for proton range QA at both clinical and FLASH dose rates in a low-cost, compact and modular package. The use of plastic scintillator provides near water-equivalent ranges (calibrated to WET with a one-time measurement) and the fast readout/live fitting capabilities reduce the time taken for range QA to the time taken for delivery of each beam energy to be measured. In principle, the whole daily range QA process could be completed with the delivery of a single treatment plan stepping through each beam energy consecutively, with the QuARC running throughout delivering range results in real-time. As such, it is expected that measurement/analysis time for daily range QA to take around 1–2 minutes, with a more optimised detector package only taking a few minutes to set up before and unmount after measurement. It is worth emphasising that if daily range QA can be performed at all beam energies, it is then no longer necessary for more comprehensive range QA protocols on a monthly or yearly basis.

The compact QuARC prototype developed for the Clatterbridge Cancer Centre demonstrated the detector’s capability to measure the range of spread-out Bragg peaks (12), which could become increasingly relevant for modern PBT facilities given the promising results with the delivery of passively scattered FLASH proton beams using ridge filters (54, 55).

### 5.3 Further work

The next stages of the development of the QuARC will be to optimise the scintillator sheet light output and improve overall robustness. It is expected that the use of polished scintillator will improve the accuracy of the calibration process measured depth-light curves by increasing sheet internal reflection. Investigation can then also be made into the applicability of the shoot-through calibration process across multiple detector modules, to isolate the cause of the observed stepping in light levels between modules. Further work is required to fully determine the dose rate dependence of the QuARC, which will need to performed at a centre with finer control of the beam nozzle current for a selection of beam energies.

The mechanical setup of the device will also be improved by reducing the size of the detector enclosure to a design that allows for the detector to be mounted onto the gantry nozzle itself, similar to the enclosure for the compact version of the QuARC (12). This will also serve to reduce the overall range uncertainty of the detector by mitigating angular misalignment. Future revisions of the front-end circuit boards will facilitate connections via USB-C rather than bespoke Molex ribbon cables, which will be off-the-shelf and make it easier to connect/disconnect boards without altering the photodiode-scintillator coupling. The FMC daughterboard will be redesigned to instead connect via the more compact Zmod (SYZYGY) FPGA interface, which enables the relatively large and excessive Nexys Video to be replaced with the more compact USB104 FPGA board (56). To improve the live-fitting speed, work will be undertaken to deploy fitting routines on the FPGA itself to make use of its additional parallelisation capabilities.

To expand the scope of the QuARC beyond on-axis range measurements and move towards a system for more comprehensive beam QA, the range reconstruction dependence on the lateral position of the beam in the detector must be determined. While the CMOS-based QuARC prototype demonstrated range stability with proton beam spot position (28), the smaller light collection area of the photodiodes warrants further investigation into the range stability of the QuARC with respect to these variables. This can be supported by Monte-Carlo simulation of the new detector geometry. Future experiments will also endeavour to experimentally measure a value of Birks’ constant for the scintillator used in the QuARC, to facilitate investigation into whether fixing this parameter can improve the range accuracy and stability of the detector. This will also investigate the stability of this parameter with external parameters such as beam energy, scintillator age and temperature.

While primarily intended for protons, which make up the vast majority of particle therapy centres active today, the QuARC can be used in principle to perform range measurements for ion beams like helium and carbon. Unfortunately, the Bortfeld model (which underpins the Kelleter model used in this analysis) does not accurately describe the increased nuclear interactions (e.g. fragmentation) (57) with such species and the increased scintillator light quenching from the higher LET heavy ions means that Birks’ first-order approximation to describe quenching also breaks down (58). One proposed solution to this limitation is to instead adopt a numerical approach to fit measured depth-light data, such as that described in (59), and expand the description of scintillator light quenching to second-order (60). Finally, using principles demonstrated in (61), future work from the group will investigate the use of the QuARC in conjunction with a scintillator fibre-based profile monitor placed in front of the QuARC. Combining 2D proton spot position measurements with 1D range measurements from the QuARC will enable 3D characterisation of proton beams, expanding the scope of the device for more comprehensive beam QA and integrated-mode proton imaging.

## 6 Conclusion

The design and performance of a full-size prototype of the Quality Assurance Range Calorimeter has been presented. The detector uses 4 32-sheet modules daisy-chained together to provide real-time measurements of proton range across the full clinical energy spectrum. The range accuracy of the detector was found to be within 0.5 mm after calibration of the depth-axis under the assumption of a uniform detector response with energy. In addition, the detector was found to be able to measure proton beams with nozzle currents of up to 50 nA without modification, with the fitted ranges agreeing with reference values to within the detector range uncertainty. Significant issues with detector calibration were observed, which will be improved in future iterations paying specific attention to optimising the scintillator light output. Overall, the QuARC presents a promising solution for fast, easy proton range QA at both clinical and FLASH regimes.

## Data Availability

The raw data supporting the conclusions of this article will be made available by the authors, without undue reservation.
