# Correction: Range quality assurance measurements for clinical and FLASH proton beam therapy using the quality assurance range calorimeter

**DOI:** 10.3389/fonc.2026.1824636

**Published:** 2026-04-24

**Authors:** Saad Shaikh, Sonia Escribano-Rodriguez, Raffaella Radogna, Ruben Saakyan, Sam Manger, Nicholas Henthorn, John-William Warmenhoven, Michael Taylor, Simon Jolly

**Affiliations:** 1 Department of Medical Physics and Biomedical Engineering, University College London, London, United Kingdom; 2 Department of Physics and Astronomy, University College London, London, United Kingdom; 3 Department of Physics, University of Bari, Bari, Italy; 4 Istituto Nazionale di Fisica Nucleare, Sezione di Bari, Bari, Italy; 5 Division of Cancer Sciences, Faculty of Biology, Medicine and Health, The University of Manchester, Manchester, United Kingdom; 6 The Christie NHS Foundation Trust, Manchester, United Kingdom

**Keywords:** proton therapy, FLASH, range quality assurance, photodiode, plastic scintillator

Affiliation The Christie NHS Foundation Trust, Manchester, United Kingdom was erroneously given as The Christie NHS Foundation Trust, Manchester Academic Health Science Centre, Manchester, United Kingdom.

Author Sam Manger, Nicholas Henthorn, John-William Warmenhoven, and Michael Taylor were erroneously assigned to affiliation Division of Cancer Sciences, Faculty of Biology, Medicine and Health, The University of Manchester, Manchester, United Kingdom. This affiliation has now been removed for authors Sam Manger, Nicholas Henthorn, John-William Warmenhoven, and Michael Taylor.

Affiliation The Christie NHS Foundation Trust, Manchester, United Kingdom was omitted for authors Sam Manger, Nicholas Henthorn, John-William Warmenhoven, and Michael Taylor. This affiliation has now been added for authors Sam Manger, Nicholas Henthorn, John-William Warmenhoven, and Michael Taylor.

Affiliation Istituto Nazionale di Fisica Nucleare, Sezione di Bari, Bari, Italy was omitted for author Raffaella Radogna. This affiliation has now been added for author Raffaella Radogna.

The original version of this article has been updated.

